# Novel Insights into the Human Gut Microbially Conjugated Bile Acids: The New Diversity of the Amino Acid-Conjugated Derivatives

**DOI:** 10.1021/acs.jafc.5c03548

**Published:** 2025-07-24

**Authors:** Carlos J. Garcia, Rocio García-Villalba, Maria D. Frutos-Lisón, David Beltrán, María Antonia Martínez-Sánchez, María Ángeles Núñez-Sánchez, Bruno Ramos-Molina, Francisco A. Tomás-Barberán

**Affiliations:** a Quality, Safety and Bioactivity of Plant-Derived Foods, Centro de Edafología y Biología Aplicada del Segura-Consejo Superior de Investigaciones Científicas (CEBAS-CSIC), Murcia 30100, Spain; b Obesity, Diabetes and Metabolism Research Group, Biomedical Research Institute of Murcia-Pascual Parrilla (IMIB-PP), Murcia 30120, Spain

**Keywords:** bile acids, MCBAs, gut microbiota, metabolomics, MS/MS spectra, GABA conjugates

## Abstract

Bile acids (BAs) are biomolecules involved in lipids and glucose metabolism, and recently discovered microbially conjugated BAs (MCBAs) play an important role. Although the production of MCBAs amidated with amino acids (AA), similarly to those hepatically produced, has been confirmed, new structural isomers, conjugated with non-proteinogenic AA, remain unidentified. This study evaluates the production of MCBAs by human gut microbiota and discriminates, for the first time, between structural isomers. Thirteen MCBAs composed of lithocholic acid conjugated either with the AA valine and leucine or the non-proteinogenic AA, 5-amino valeric acid, and different aminobutyric acid derivatives (GABA and 2-amino-butyric) were confirmed by MS/MS fragmentation patterns and authentic standards. Only the fragmentation patterns in positive polarity confirmed the occurrence of different amino acid-conjugated derivatives. This study showed the microbiota’s ability to produce MCBAs from both proteinogenic and non-proteinogenic AAs and disclosed, for the first time, the MS fragmentation rules to differentiate the structural isomers of MCBAs with amino groups at different positions. The MCBA content in fresh stool samples was 3-fold that of hepatically conjugated BAs, confirming their relevance. These findings will introduce the methodology for the analysis of this new MCBA family in BA analysis.

## Graphical abstract

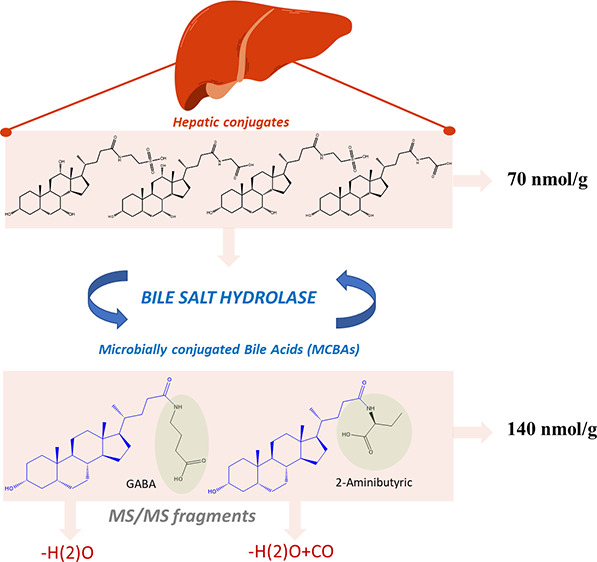

## 1 Introduction

Bile acids (BAs) are key endogenous steroids essential for human health.^1^ They aid in fat digestion and absorption due to their amphipathic nature and function as metabolic regulators by activating nuclear and G protein-coupled receptors, influencing hepatic lipid and glucose homeostasis.^2,3^ BAs are synthesized in hepatocytes from cholesterol, producing the primary BAs cholic acid (CA) and chenodeoxycholic acid (CDCA), which are then conjugated with glycine or taurine by bile acid CoA amino acid *N*-acyltransferase (BAAT) and stored in the gallbladder.^4,5^ Upon release into the intestine, gut bacteria bearing bile salt hydrolase (BSH) activity deconjugate them, enabling their transformation into secondary BAs. This interaction with the gut microbiota results in a variety of secondary BAs, including dehydroxylated, oxidized, and epimerized forms, and remains a significant area of research in pathophysiology.^6,7^ The discovery of microbially conjugated bile acids (MCBAs) through reconjugation with amino acids has expanded our understanding of BAs diversity and its biological implications.^8,9^ Additionally, a recent study has reported that BSH possesses dual functions in BA metabolism including an unknown role as an amine *N*-acyltransferase that conjugates amines to BAs. Initially, MCBAs referred to amides, or amidated MCBAs, conjugated at the C24 acyl site, akin to conjugation with glycine and taurine in hepatocytes with amino acids.^10^ Recently, we identified other isomers of these compounds and we suggested a novel BA reconjugation mechanism based on esterification reactions to produce esterified MCBAs, although this identification has not been confirmed so far.^11^ This suggests the occurrence of a new class of conjugates, underscoring the need for detailed investigation into the distinctions between different MCBAs due to their overlooked significance and potential impact on metabolic disorders.

The mechanisms of production and biological effects of these newly identified amidated MCBAs are not yet fully understood. Previous studies have suggested that the occurrence of MCBAs might represent a bacterial strategy to mitigate the toxicity of unconjugated BAs, facilitated by the high activity of BSH from anaerobic intestinal bacteria including *Bacteroides*, *Clostridium*, *Lactobacillus*, and *Bifidobacterium* species.^12^ Moreover, BAs, both general and conjugated, have been correlated with significant metabolic disorders such as dyslipidemia, hypercholesterolemia, and dysglycemia.^13–16^ This underscores the importance of understanding the balance between conjugated and unconjugated BAs in the ileum and their interactions with intestinal receptors. These interactions influence insulin secretion and modulate intestinal FXR-FGF15 signaling, potentially reducing hepatic cholesterol levels and decreasing lipogenesis.^17–19^

It has been suggested that there may be hundreds of novel BA conjugates with amino acids and non-proteinogenic amino acids. However, their characterization remains challenging as many of them are isomers. Analytical platforms combining liquid chromatography, ion mobility spectrometry, and mass spectrometry (LC–IMS–MS) have been employed to separate and classify these compounds, typically using cholic acid as the molecular core.^20^ Despite differences in retention times, isomeric forms can still be difficult to distinguish due to their similar MS/MS spectra. Semiempirical MS/MS libraries have been developed for BAs conjugated with 18 common amino acids to explore fragmentation patterns, using lithocholic acid, deoxycholic acid, and cholic acid as core structures.

However, these studies did not account for positional and stereoisomers of the BAs, as current MS/MS techniques are unable to differentiate them. Similarly, the position of the amino group in the conjugated amino acids was not considered and these isomers would share the same *m*/*z* and likely have very similar MS/MS spectra.^21^ This limitation is particularly relevant when trying to distinguish between isomers of conjugates with amino acids or non-proteinogenic amino acids, for example, valine and 5-aminovaleric acid, or γ-aminobutyric acid and its structural isomers 2- and 3-aminobutyric acid. Addressing these challenges is crucial to deciphering subtle differences in fragmentation patterns and improving structural elucidation.

This study aims to refine a bioanalytical method by LC-MS for analyzing the new family of MCBAs in *in vitro* fecal incubations and in fresh stool samples, thoroughly investigating their MS/MS fragmentation patterns. This will significantly expand the understanding of this new family of reconjugated BAs, which have not been previously considered. We aim to deepen our knowledge of BAs as signaling and metabolic regulatory molecules, to address their links to metabolic disorders, bowel disease, and cancer.^3,22–29^ This research seeks to identify new isomers of MCBAs, further expanding the scope of recently discovered MCBAs, which should be considered in future research. In this way, a new field of research is opened, once again highlighting intestinal microbiota as an increasingly important variable to consider in the study of BAs.

## 2 Materials and Methods

### 2.1 Chemicals

Acetonitrile and water 0.1% (v/v) formic acid were purchased from J.T. Baker (Deventer, The Netherlands), and formic acid was obtained from Panreac (Barcelona, Spain). Authentic standards of 3,7-dihydroxy-5-cholan-24-oic acid (chenodeoxycholic acid) and 3-hydroxy-11-oxo-5-cholan-24-oic acid (3-oxo-chenodeoxycholic acid) were purchased from Avanti Polar Lipids (Alabaster, Alabama, USA). The *N*-(3α,7α,12α-trihydroxy-5β-cholan-24-oyl)-glycine (glycocholic acid), *N*-(3α,7α,12α-trihydroxy-5β-cholan-24-oyl)-taurine (taurocholic), 3α,7α,12α-trihydroxy-5β-cholan-24-oic acid (cholic acid), 3α,7β,12α-trihydroxy-5β-cholan-24-oic acid (ursocholic), 3α,6β-dihydroxy-5β-cholan-24-oic acid (murideoxycholic acid), 3α,6α-dihydroxy-5β-cholan-24-oic acid (hyodeoxycholic acid), 3α,7β-dihydroxy-5β-cholan-24-oic acid (ursodeoxycholic acid), 3α,12α-dihydroxy-5β-cholan-24-oic acid (deoxycholic acid), 3α-hydroxy-5β-cholan-24-oic acid (lithocholic), and 3β-hydroxy-5β-cholan-24-oic acid (isolithocholic) were purchased from Cayman Chemical (Ann Arbor, Michigan, USA). Taurocholic acid, taurochenodeoxycholic acid, taurodeoxycholic acid, taurolithocholic acid, glycochenodeoxycholic acid, glycodeoxycholic acid, cholic acid-2,2,3,4,4-d5, taurocholic acid-d5, and tauroursodeoxycholic acid were purchased from Sigma-Aldrich (Darmstadt, Germany). The microbially conjugated bile acids (MCBAs) valolithocholic acid, valolithocholate ester, leucolithocholic acid, leucolithocholate ester, 4-aminobutyric lithocholic acid, and 4-aminobutyric lithocholate ester were synthesized by Eurofins Villapharma Research (Parque Tecnológico de Fuente Álamo, Ctra. El Estrecho-Lobosillo, Km. 2,5- Av. Azul 30320 Fuente lamo de Murcia, Spain).

### 2.2 Collection of Human Fecal Samples

Seventeen healthy donors provided stool samples for this study: 11 volunteers were recruited from the Biomedical Research Institute of Murcia (IMIB) and six from the Centro de Edafología y Biología Aplicada del Segura (CEBAS-CSIC, Murcia, Spain). All experiments were performed in accordance with the ethical guidelines outlined in the Declaration of Helsinki and approved by the CSIC ethics committee. Informed consents were obtained from human participants of this study. The stool samples from the six volunteers recruited at CEBAS-CSIC were used for *in vitro* incubations because their samples had been previously characterized in similar studies of microbial BA metabolism. These secondary BAs and MCBAs identified *in vitro* were evaluated in the stool samples from all 17 donors by untargeted analysis using UPLC-ESI-QTOF-MS. In addition, the stool samples of the 11 volunteers recruited at IMIB were analyzed for targeted quantification of primary and secondary BAs using methodology by *UHPLC-QqQ-MS* (Figure S1).

### 2.3 *In Vitro* Incubation of Fecal Samples

Preparation of fecal suspensions and subsequent fermentation experiments were performed similarly to previous studies with brief modification.^10^ The fermentation procedure was performed under anoxic conditions in an anaerobic chamber (Concept 400, Baker Ruskinn Technologies, Ltd., Bridgend, South Wales, UK) with an atmosphere consisting of N_2_/H_2_/CO_2_ (85:5:10) at 37 °C. Aliquots of stool samples (10 g) were diluted 1/10 w/v in Nutrients Broth supplemented with 0.05% l-cysteine hydrochloride and homogenized by a stomacher in filter bags. Aliquots of fecal suspensions (50 μL) of each volunteer were inoculated into 5 mL of fermentation medium anaerobe Wilkins Chaldean containing 50 μM chenodeoxycholic acid (CDCA) or lithocholic acid (LCA). The CDCA was selected because it is a primary BA with medium hydrophobicity and is used by the gut bacteria to produce the secondary BAs. In previous studies, bacteria converted CDCA into LCA, which is more hydrophobic and cytotoxic, and then the gut microbes reduce its hydrophobicity by reconjugation with amino acids. The LCA was used to confirm whether the MCBAs are produced from the LCA or from its isomers or epimers. Three replicate cultures were prepared in parallel from each fecal suspension. Samples were collected at 0, 24, 48, 72, and 96 and after 120 h (5 days) of incubation at 37 °C. The duration of the fecal incubation was set in order to ensure conversion procedures by the gut microbiota. Usually after 24–48 h, the bacteria are already in a stationary phase and it is in this phase when the secondary metabolism, which acts in this conversion, commonly occurs.^31^ Two control samples were prepared: fermentation medium with fecal inoculum but without BAs and fermentation medium with BAs but in the absence of bacteria. In addition, fecal samples of these volunteers were also directly analyzed without incubation.

### 2.3 Analysis of Bile Acids

#### 2.3.1 Analysis of Primary and Secondary Bile Acids by UHPLC-QqQ-MS

Samples (approximately 5 mg of lyophilized dry fecal matter) were placed in a 2 mL Eppendorf tube and mixed with 20 μL of internal standard, 800 μL of 0.1 M NaOH, and a steel bead. Samples were agitated with a bullet blender for 3 min at speed 8 and vortexed for 5 min. Samples were subsequently incubated at 60 °C for 1 h. A volume of 600 μL of water was added, and the samples were centrifuged for 10 min at 15,000 rpm and 4 °C. Supernatants were transferred to a new tube and centrifuged one more time. The resulting supernatants were loaded to an SPE cartridge (Oasis HLB 30 mg sorbent) previously conditioned with 1 mL of methanol and 1 mL of water. Cartridges were washed with 1 mL of water, 1 mL of hexane, and 1 mL of water. Then, cartridges were dried under high vacuum, and compounds were eluted with 500 μL of methanol twice. The eluates were evaporated in a SpeedVac at 45 °C. Samples were reconstituted with 100 μL of methanol and transferred to glass vials for analysis.

The chromatographic separation was performed on a Kinetex EVO C18 (150 mm × 2.1 mm) column. Mobile phase A was 0.1% ammonium hydroxide 10 mM ammonium acetate, and mobile phase B was acetonitrile. The column temperature was set at 27 °C, the flow was 0.5 mL min^–1^, and the injection volume was 2 μL. The gradient started with 25% B; increased in 7.2 min to 30% B, in 13.2 min to 50% B, and in 13.5 min to 100% B; held for 1.5 min and decreased to the initial conditions in 15.5; and finally held for 2 min up to 17.5 min.

#### 2.3.2 Untargeted Metabolomics Analysis of Secondary BAs and MCBAs by UPLC-ESI-QTOF-MS

Fresh fecal samples and samples after incubation were extracted prior to analysis by UPLC-ESI-QTOF-MS. The samples were extracted according to previous studies with modification in the case of the liquid samples after 5 days of in vitro incubation (Figure S2).^32^ Incubation solution (5 mL) was vortexed and centrifuged at 4500 rpm for 15 min at 4 °C. Then, an SPE extraction was performed using a cartridge HyperSep Sep 500 mg/2.8 mL C18 by using manifolds. The cartridge was then washed with water (5 mL), and BAs were eluted with ethanol (5 mL).

The metabolomics analysis was performed on a U-HPLC instrument (Infinity 1290; Agilent) coupled to a high-resolution mass spectrometer with a quadrupole time-of-flight mass analyzer (6550 iFunnel Q-TOF LC/MS; Agilent) with an Agilent Jet Stream (AJS) electrospray (ESI) source. The mass analyzer was operated in negative mode under the following conditions: gas temperature 150 °C, drying gas 14 L/min, nebulizer pressure 40 psig, sheath gas temperature 350 °C, sheath gas flow 11 L/min, capillary voltage 3500 V, fragmentor voltage 120 V, and octapole radiofrequency voltage 750 V. Data were acquired over the *m*/*z* range of 50–1700 at the rate of 3 spectra/s. The *m*/*z* range was autocorrected on reference masses 112.9855 and 1033.9881.

The MS/MS target product ion spectra were acquired at *m*/*z* 100–1100 using a retention time window of 1 min, a range of 5–60 eV of collision energy, and an acquisition rate of 1 spectra/s. The chromatographic analysis was performed with a reversed-phase C18 column (Poroshell 120, 3 mm × 100 mm, 2.7 μm pore size) at 30 °C, using water + 0.1% formic acid (phase A) and acetonitrile + 0.1% formic acid (phase B) as mobile phases with a flow rate of 0.4 mL/min. The gradient started with 50% B, increased in 4 min to 90% B, in 3 min to 99% B, held for 3 min, and decreased to the initial conditions during 3 min. The injection volume for all samples was 3 μL. Raw data acquired was processed by MS-DIAL 5.1.2 (prime.psc.riken.jp/compms) through the application of a featured extraction based on the in-house database built for bile acids. After data processing, the BAs identified were analyzed by Mass Hunter Qualitative 10.0 qualitative (Version B.10.0, Agilent software metabolomics, Agilent Technologies, Waldbronn, Germany).

#### 2.3.3 Graphics Software

GraphPad Prism software (version 10.2.1) was used for statistical analyses and for generating all graphs presented in this manuscript. A *t* test was performed to assess statistical differences among the BA groups shown in Figure 6e.

## Results

The analytical method proposed in this study was designed to maximize the extraction of the MCBAs and to distinguish among the structural isomers of the different MCBAs identified. To this goal, the extraction, chromatography, and MS parameters were especially set to increase the equipment response to these BAs. This study evaluated the bacterial metabolism of CDCA and LCA during 120 h to produce the secondary BAs via the classical mechanisms as well as the novel MCBAs.

### Identification of the Secondary BAs in *In Vitro* Production

As previously described, total fecal bacteria incubated with CDCA primarily produced ursodeoxycholic acid (UDCA), isoursodeoxycholic acid (isoUDCA), lithocholic acid (LCA), and its epimer (iLCA). Among these, LCA and iLCA accumulated significantly after incubation for 5 days of incubation. The fermentation results revealed a global trend toward the accumulation of monohydroxylated BAs and CDCA epimers, predominantly via metabolism at the 7α-hydroxyl position (Figure 1, red box), rather than the 3α position (Figure 1, gray box). The inclusion of 24 h sampling intervals allowed for the identification of pathway intermediates (Figure 2). The secondary BAs derived from the bacterial metabolism of CDCA included the following: (i) dehydroxylated BAs such as LCA, (ii) oxo-BAs such as 7-oxoLCA, 3-oxoCDCA, 3-oxo-5β-cholan-24-oic acid, and 7-oxo-5β-cholan-24-oic acid; and (iii) epimeric BAs such as isoCDCA, UDCA, isoUDCA, and isoLCA (Table 1). At time 0, CDCA, LCA, isoLCA, 3-oxo-chenodeoxycholic acid, 7-oxo-lithocholic acid, 3-oxo-5β-cholan-24-oic acid, and isochenodeoxycholic acid were detected in fecal samples. Regarding dehydroxylated BA production, LCA levels increased during incubation. However, 7α-5β-cholan-24-oic acid and its epimer were not detected, suggesting rapid conversion of 7α-5β-cholan-24-oic acid to 7-oxo-5β-cholan-24-oic acid. Additionally, the abundance of 7-oxo-5β-cholan-24-oic acid was approximately 100 times lower than that of 3-oxo-5β-cholan-24-oic acid. For oxo-BAs, 7-oxo-lithocholic acid levels increased after 24 h of incubation. In contrast, 3-oxo-CDCA levels decreased during CDCA fermentation (Figure 2), suggesting that its production from CDCA was negligible. Instead, 3-oxo-CDCA derivatives (Figure 1, gray box) were primarily produced from fecally excreted 3-oxo-CDCA. Epimeric BA production was more prominent via metabolism at the 7α-hydroxyl position (Figure 1, red box). The most abundantly produced epimers were UDCA, isoLCA, and isoUDCA, along with LCA, after incubation (Figure 1, blue molecules). However, the epimer isochenodeoxycholic acid, derived from 3-oxo-chenodeoxycholic acid, increased after 24 h of incubation and then decreased. This finding suggests that isoCDCA acid was primarily utilized for isoUDCA production, emphasizing the importance of secondary BA production via the 7α-hydroxyl position. The secondary BAs most abundantly produced during incubation, LCA and isoLCA, and their precursors, UDCA and isoUDCA, exhibited similar kinetic patterns (Figure 2, red lines). These compounds increased rapidly within the first 24–48 h of incubation. While LCA and isoLCA stabilized after 48 h, UDCA and isoUDCA peaked at 24 h and subsequently declined. These results suggest that MCBA production or a significant increase in their levels could begin between 24 and 48 h, as LCA and isoLCA serve as the BA backbones for MCBA synthesis. During LCA incubation, no secondary BAs were identified as products of the LCA bacterial metabolism. Instead, LCA levels decreased progressively throughout the sampling period, indicating that other possible metabolites could be appearing.

**Figure 1:**
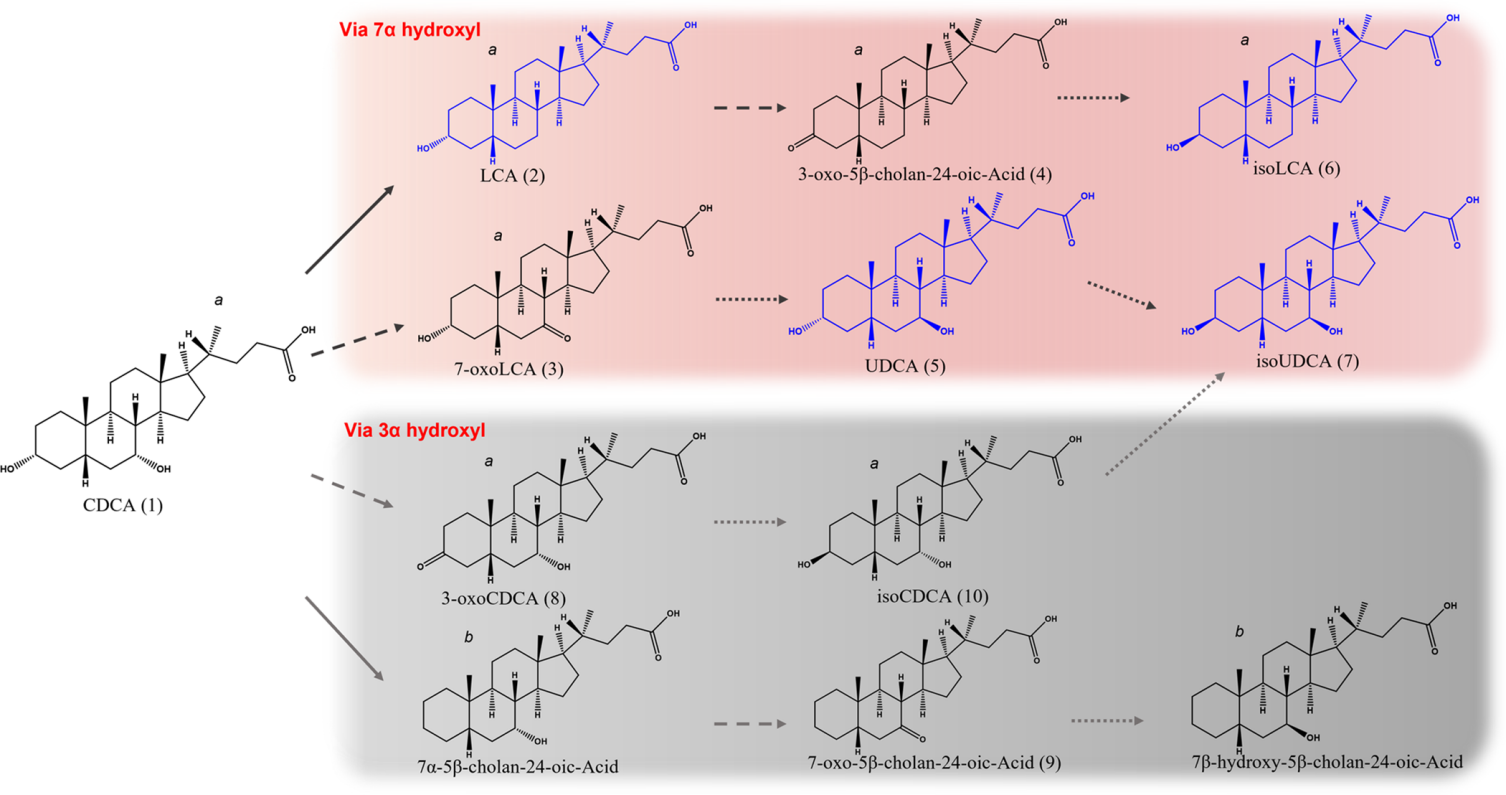
Global trend of CDCA bacterial metabolism. Red box: CDCA bacterial metabolism of 7α hydroxyl; gray box: CDCA bacterial metabolism of 3α hydroxyl. (1) Chenodeoxycholic acid; (2) lithocholic acid; (3) 7-oxo-lithocholic acid; (4) 3-oxo-5β-cholan-24-oic-acid; (5) ursodexycholic acid; (6) isolithocholic acid; (7) isoursodexycholic acid; (8) 3-oxo-chenodeoxycholic acid; (9) 7-oxo-5β-cholan-24-oic-acid; (10) isochenodeoxycholic acid. Solid arrow: bacterial enzymatic activity of 3/7α dehydroxylase; dashed arrow: bacterial enzymatic activity of 3/7α hydroxysteroid dehydrogenase (7/3 α/β-HSDH); bullet arrow: bacterial enzymatic activity of 3/7 α/β hydroxysteroid dehydrogenase (7/3 α/β-HSDH). Blue BA molecules: bile acids accumulated produced after the incubation; black BA molecules: bile acids identified during the incubation. (a) Identified in fecal samples before incubation; (b) not identified.

**Figure 2:**
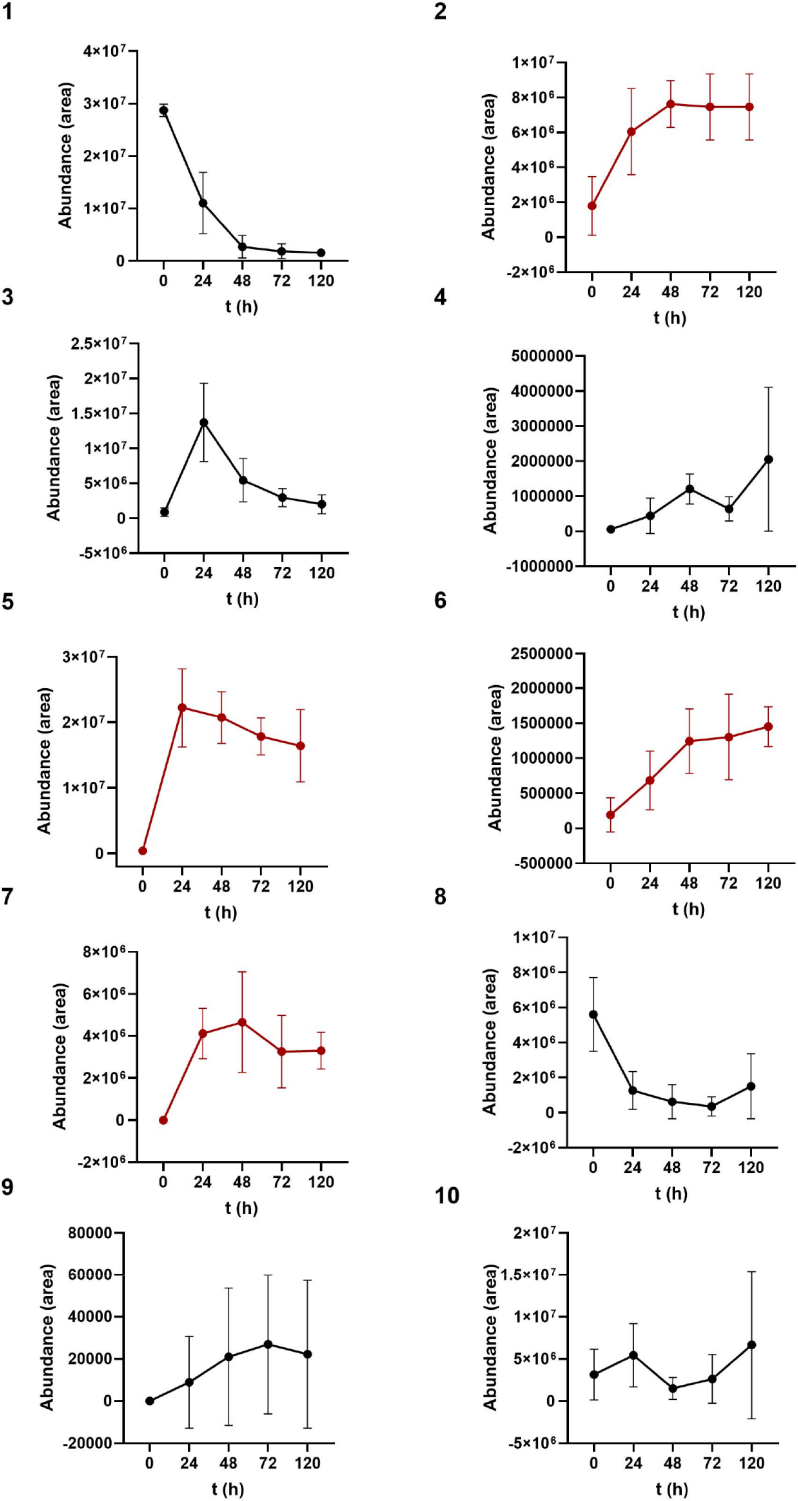
Production kinetics of BAs during 120 h. Graphs 1–7 correspond to BAs produced via 7α hydroxyl position and 8, 9, and 10 to BAs produced via 3α. Graphs with red lines (2, 5, 6, 7) correspond to BAs mostly produced. *N* = 6 volunteers at each sampling point were included. BA graphs: (1) chenodeoxycholic acid; (2) lithocholic acid; (3) 7-oxo-lithocholic acid; (4) 3-oxo-5β-cholan-24-oic-acid; (5) ursochenodexycholic acid; (6) isolithocholic acid; (7) isoursochenodexycholic acid; (8) 3-oxo-chenodeoxycholic acid; (9) 7-oxo-5β-cholan-24-oic-acid; (10) isochenodeoxycholic acid.

**Table 1:** Primary and Secondary Bile Acids Identified and Confirmed with Authentic Standards and MS/MS Fragmentation Patterns in the *In Vitro* Study

ID	compound name	formula	*m*/*z*	ppm	Rt	MS/MS fragments (neg polarity)	collision E
**1**	chenodeoxycholic acid (CDCA)^a^	C_24_H_40_O_4_	391.2859	1.32	3.4	391.2862; 373.2750	40
**2**	lithocholic acid (LCA)^a^	C_24_H_40_O_3_	375.2922	4.6	5.18	375.2939; 357.2838; 355.2669	50
**3**	7-oxo-lithocholic acid (7-oxoLCA)	C_24_H_38_O_4_	389.2712	3.76	2.8	389.2722; 345.2808; 343.2657	40
**4**	3-oxo-5β-cholan-24-oic-acid	C_24_H_38_O_3_	373.2751	0.75	5.35	373.2737; 355.2634	30
**5**	ursodexycholic acid (UDCA)	C_24_H_40_O_4_	391.2851	–0.72	2.37	391.2859; 373.2780	40
**6**	isolithocholic acid (isoLCA)	C_24_H_40_O_3_	375.2916	3.01	4.5	375.2920; 357.2808; 355.2650	50
**7**	isoursodexycholic acid (isoUDCA)	C_24_H_40_O_4_	391.2849	–1.23	2.2	391.2855; 373.2784	40
**8**	3-oxo-chenodeoxycholic acid (3-oxoCDCA)^a^	C_24_H_38_O_4_	389.2711	3.5	3.68	389.2708; 345.2813; 343.2664; 371.2643	40
**9**	7-oxo-5β-cholan-24-oic-acid	C_24_H_38_O_3_	373.2759	2.89	4.69	373.2740; 355.2638	30
**10**	isochenodeoxycholic acid (isoCDCA)	C_24_H_40_O_4_	391.2850	–0.98	2.73	391.2858; 373.2755	40

a Detected at time 0.

### *In Vitro* Evaluation of the Microbial Production of Reconjugated BAs (MCBAs)

In addition to the previously known transformations, recent studies have shown a new set of MCBAs formed by reconjugation with amino acids. In this study, the bacterial reconjugation of secondary BAs was studied after the incubation with CDCA. An untargeted metabolomic approach was applied by searching for a list of possible new microbial metabolites formed by a BA backbone (CDCA, LCA) conjugated with different AAs naturally occurring in humans. Samples were analyzed by MS and MS/MS fragmentation patterns in both negative and positive polarities. A wide range of collision energies (10–60 eV) were used for screening all fragmentation possibilities as well as a combination of collision energies and fragmentor voltage of the electrospray ionization source. In negative mode, different isomers were identified for the *m*/*z* values 488.3745 corresponding to C_30_H_51_NO_4_ (leucine conjugated with LCA), 474.3589 corresponding to C_29_H_49_NO_4_ (valine conjugated with LCA), and 460.3432 corresponding to C_28_H_47_NO_4_ (aminobutyric acid conjugated with LCA). In addition, the MS/MS fragmentation pattern in negative mode was able to confirm conveniently the presence of this MCBAs because it released the nitrogenous acid residue as the major fragments: *m*/*z* 116.0717 for valine conjugates, 130.0873 for leucine conjugates, and 102.0560 for aminobutyric acid conjugates, using the range from 20 to 50 eV.^10,20,21^ The fragmentation study in negative mode also showed characteristic fragments of the MCBAs based on lithocholic acid molecules such as the neutral loss of carbon dioxide (*m*/*z* 430.3670 in the case of valine conjugates). As can be observed for valine conjugates, in negative polarity, all the isomers showed the same fragmentation patterns and it was not possible to discriminate between them (Figure 3). The most common conjugation with amino acids previously reported is the amidation at the 24-acyl site, but the large number of isomers suggests that other types of bonds or compounds could exist.^8^ Previous studies have suggested the formation of novel MCBAs by esterification reactions.^11^ This led us to get synthesized standards of amides and esters of lithocholic acid with leucine (leucolithocholic acid and leucolithocholate ester), valine (valolithocholic acid and valolitocholate ester), and aminobutyric acid (4-aminobutyric lithocholic acid and 4-aminobutyric lithocholic ester). In both negative and positive polarities, the synthesized amides and esters showed a similar fragmentation pattern with the main loss of the amino acid residue. However, although in negative polarity there were no fragmentation differences between the isomers, the behavior of the different isomers was discriminant in positive polarity. In addition, the synthesized esters showed retention times different from those of the candidates. These results led to the rejection of the hypothesis that these isomers were esterified MCBAs. The analysis of the standards in positive polarity showed characteristic fragments corresponding to the protonated nitrogenous acids released from the MCBA molecule (Figure 4). The conjugates with the amino group located on the α carbon (conjugates with leucine and valine) release a fragment related to water plus carbon monoxide loss of the amino group (−46) (Figure 4a,b) whereas those conjugates with the amino group in the terminal position (conjugates with 4-aminobutyric acid) release a characteristic MS/MS fragment related to the water loss (−18) of the amino group (Figure 4c). These characteristic fragments of the released amino acids allowed us to identify the different structural isomers and opened the possibility that they could be isomers conjugated with other nitrogenous acids. In the case of valine conjugates, five isomers were identified. Three of them showed a characteristic fragment at *m*/*z* 72.0776 (loss of water plus carbon monoxide from the released amino acid), indicating the presence of an amino acid with the amino group on the α carbon, and they were identified as valolithocholic acid, formed by the conjugation of valine with lithocholic acid (Figure S3). The isomer identified at a retention time of 4.6 min was confirmed with the standards as l-valolithocholic acid. The other two isomers showed a characteristic fragment at *m*/*z* 100.07 (loss of water from the released amino acid) indicating the presence of the amino group in the terminal position, and therefore they were identified as 5-aminovalolithocolic acid, formed by the conjugation of 5-aminovaleric acid with lithocholic acid (Figure S3). It was only possible to differentiate the structural isomers of both conjugates with nitrogenous acids containing the terminal amino group and those with the amino group located on the α carbon after the nitrogenous acid was released and subsequently fragmented. In the case of leucine conjugates, the three isomers showed the same fragmentation pattern with *m*/*z* 132.1012 corresponding to leucine, and *m*/*z* 86.0957 corresponding to the loss of water plus carbon monoxide from the released amino acid, and were identified as leucolithocholic acid. The isomer identified at retention time 5.01 min was confirmed with the standards as l-leucolithocholic acid (Figure S4). Regarding aminobutyric acid conjugates, five isomers were identified, three of them with the characteristic fragment at *m*/*z* 58.0599 (loss of water plus carbon monoxide from the released amino acid) and identified as 2-aminobutyric lithocholic acid, and the other two with a characteristic fragment at *m*/*z* 86.0594 (loss of water from the released amino acid) and identified as 4-aminobutyric lithocholic acid (Figure S5). The isomer at 3.6 min was confirmed by authentic standards.

**Figure 3:**
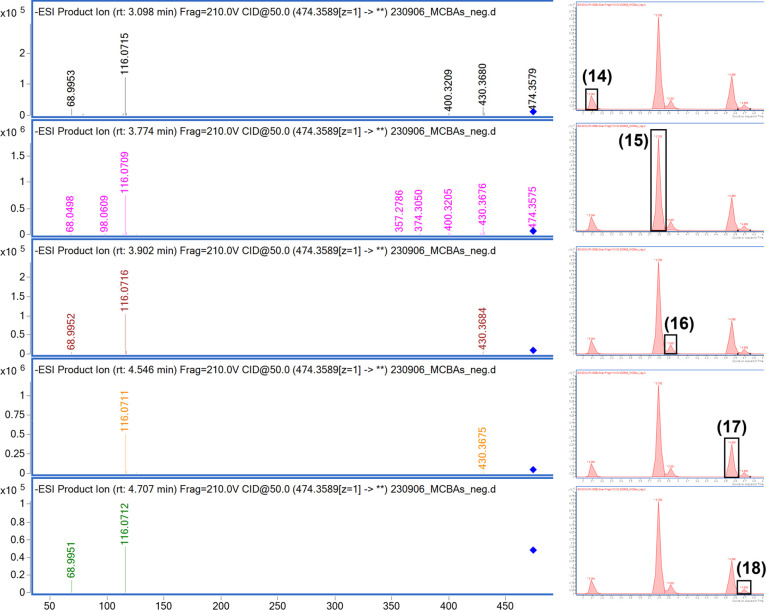
MS/MS spectral fragmentation in negative mode polarity of the MCBA isomers with a *m*/*z* corresponding to valine. 5-Aminovaloisolithocholic acid (**14**), 5-aminovalolithocholic acid (**15**), two valolithocholic acid isomers (**16**, **18**), and l-valolithocholic acid (**17**).

**Figure 4:**
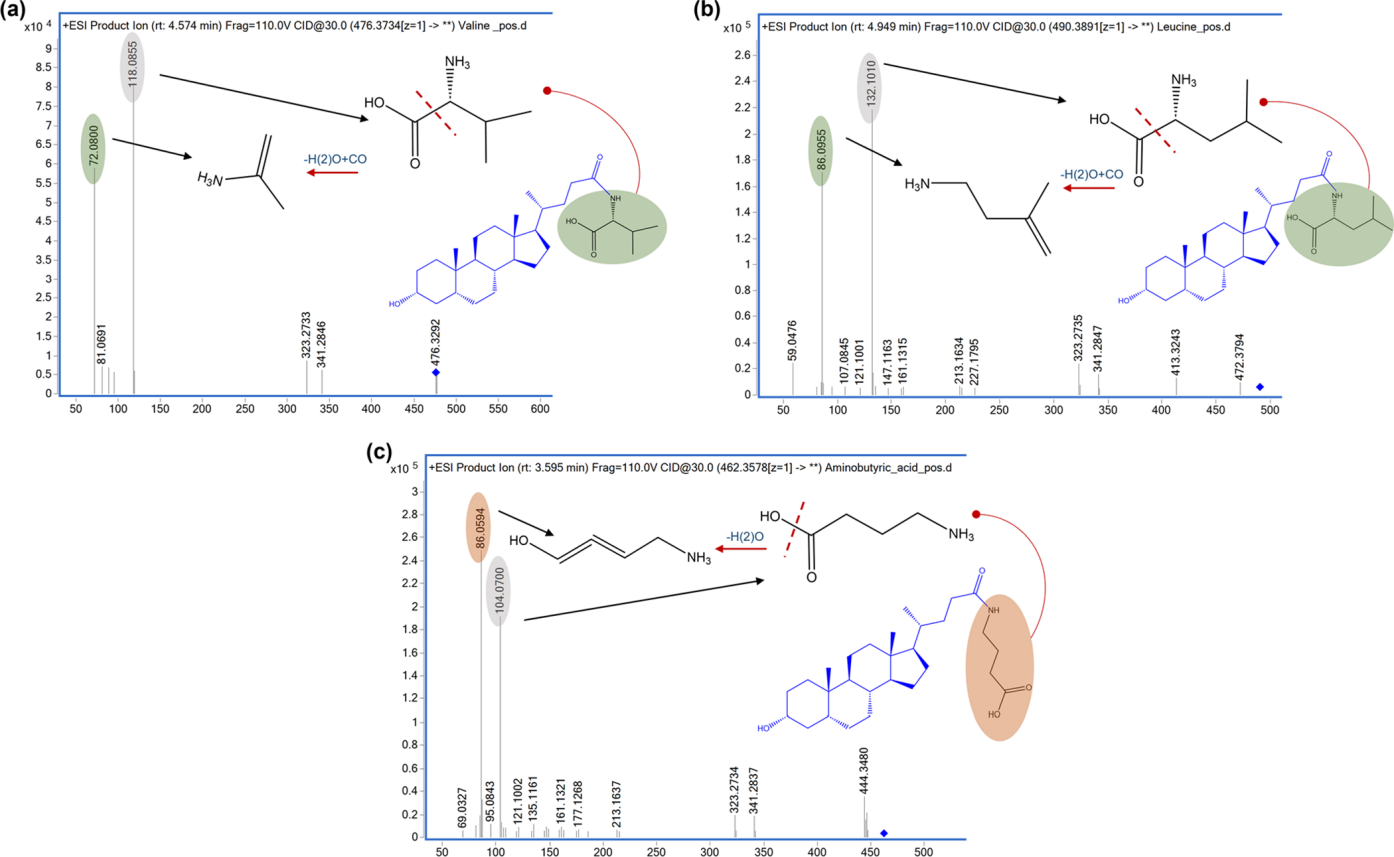
MS/MS spectral fragmentation of the MCBA derivatives from nitrogenous acids with terminal and at the α carbon amino group. (a) MS/MS spectra of MCBA derivatives from valine (nitrogenous acids with amino group at α carbon). (b) MS/MS spectra of MCBA derivatives from leucine (nitrogenous acids with amino group at α carbon). (c) MS/MS spectra of MCBA derivatives from 4-aminobutyric lithocholic acid (nitrogenous acids with terminal amino groups). Characteristic fragment of the nitrogenous acid (gray circle); characteristic fragment of H_2_O plus CO loss related to MCBAs with amino group at α carbon (green circle); and characteristic fragment of the H_2_O loss related to MCBAs with terminal amino groups (orange circle).

The fragmentation results suggested that the occurrence of the characteristic fragment of the nitrogenous acids with an amino group on the α carbon corresponding to the water plus carbon monoxide loss is more feasible because the proximity of the functional groups, which promote electronic interactions and destabilize the carboxyl group, encourages its complete elimination. In the case of nitrogenous acids with a terminal amino group, the spatial separation favors dehydration, stabilizing the remaining carbonyl functionality. This fragmentation behavior was contrasted with the fragmentation pattern of the nitrogenous acids with the amino group at terminal and the α carbon position by the Competitive Fragmentation Modeling for Metabolite Identification (CFM ID; https://cfmid.wishartlab.com/). Examining the case of the valine and 2-aminobutyric acid (amino acids with an amino group on the α carbon of the carboxyl group) and 5-aminovaleric acid and 4-aminobutyric acid (amino acids with terminal amino group), similar results were found. Depending on the position of the amino group, at the α carbon or terminal position, the main fragment will be the loss of either H_2_O+CO or H_2_O, respectively. Additionally, public MS/MS spectrometry data mining on GNPS/MassIVE (Global Natural Product Social Molecular Networking/Mass Spectrometry Interactive Virtual Environment) has been used to generate specific libraries and conduct mass spectrum queries using (MassQL) to identify new and unknown conjugated bile acids.^33^ They found thousands of MS/MS spectra with dihydroxylated and trihydroxylated bile acid queries, but they did not find specific fragments, which differentiate the MCBAs based on nitrogenous acids. In our case, the GNPS MASST search tool was used to find coincidences to differentiate between them, but we did not find these fragments. The authors noted that this is particularly effective when the MS/MS spectrum of conjugated BAs shows a significant change. This could be the reason why these fragments were not found since the injection and collision energy features used to find these fragments were unusually high for regular LC-MS methods included in libraries.

Both the conjugates with nitrogenous acids containing the terminal amino group and those with the amino group located on the α carbon showed a common fragment with *m*/*z* 341.2821 that appeared as the characteristic fragment of these molecules by the Competitive Fragmentation Modeling for Metabolite Identification (CFM ID; https://cfmid.wishartlab.com/), and another fragment with *m*/*z* 323.2722 corresponding to the loss of a water molecule from the previous fragment.

The principal MCBAs identified were secondary BAs reconjugated with the amino acid leucine, valine, and the non-proteinogenic amino acids 5-aminovaleric acid and aminobutyric acid. The LCA and isoLCA were mainly the BA backbones identified as part of MCBAs after the incubation with CDCA (Figure 5). The incubations with LCA were used to validate those MCBAs derived specifically from LCA and not from isoLCA since they have the same mass and differ only in retention time. Three leucine-derived MCBAs were identified after incubation with CDCA and LCA; two valine-derived MCBAs were identified after the incubation with LCA instead of three with CDCA; one 5-aminovaleric acid-derived MCBAs with LCA instead of two with CDCA, and two aminobutyric acid-derived MCBAs were identified after the incubation with LCA instead of five with CDCA. This made it possible to confirm the occurrence of the MCBAs derived from LCA and allowed the discrimination of some MCBAs derived from LCA and isoLCA. Other MCBA candidates based on CDCA were tentatively identified, but they were not presented in sufficient amount to be validated by MS/MS fragmentation.

**Figure 5:**
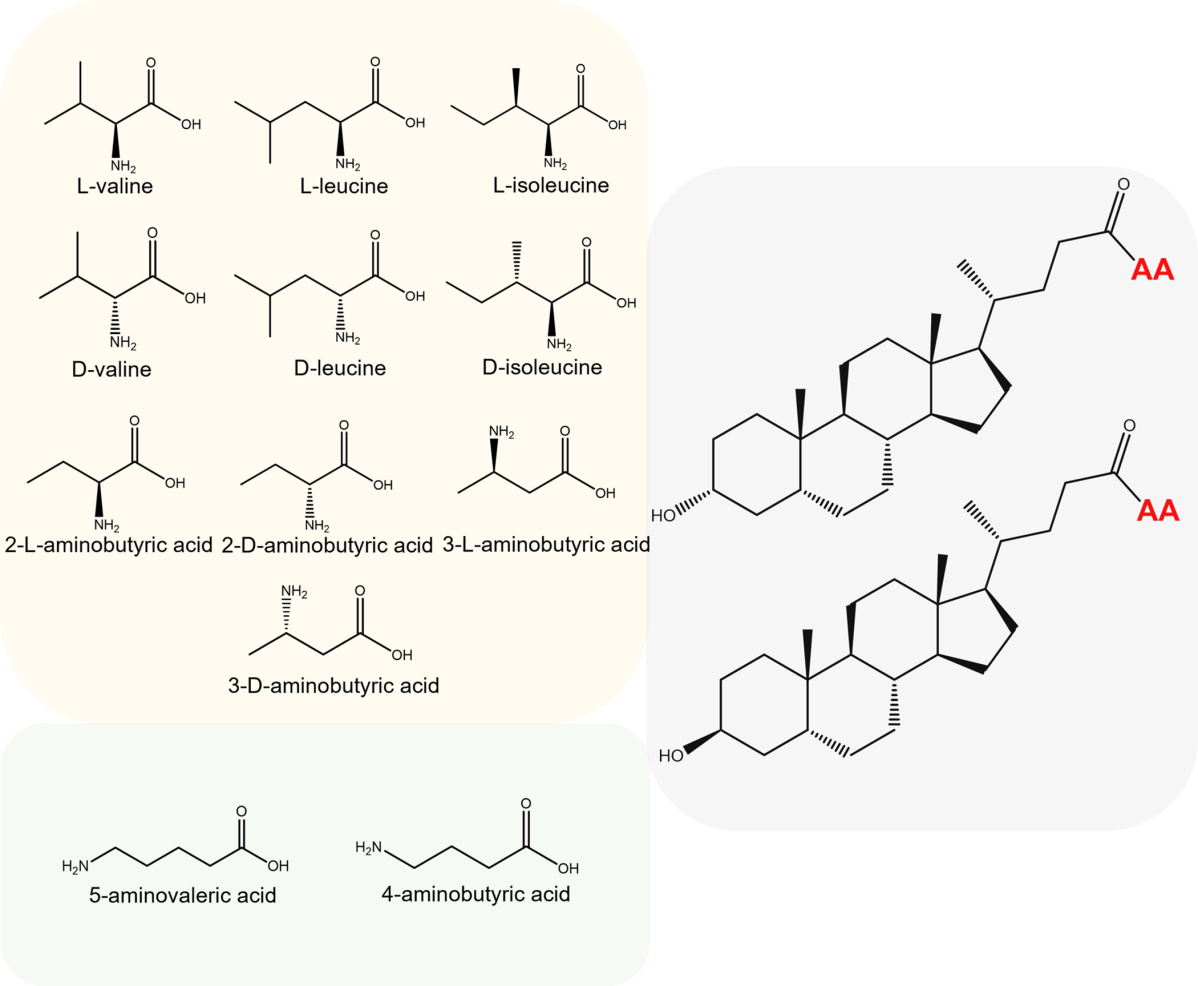
Possible combinations of MCBAs with valine, leucine, 5-aminovaleric acid, and aminobutyric acid derivatives from the lithocholic acid backbone. Nitrogenous acids with terminal amino groups (5-aminovaleric acid and 4-aminobutyric acid) (green box); Nitrogenous acids with amino groups at α carbon (orange box); lithocholic and isolithocholic acid (gray box).

Nine MCBAs conjugated with nitrogenous acids containing the amino group at the α carbon, six amino acids, and three non-proteinogenic amino acids, and four with a terminal amino group, all non-proteinogenic acids, were identified. The MCBAs identified were two leucolithocholic acid isomers (**11**, **13**), l-leucolithocholic acid (**12**), 5-aminovaloisolithocholic acid (**14**), 5-aminovalolithocholic acid (**15**), two valolithocholic acid isomers (**16**, **18**), l-valolithocholic acid (**17**), 4-Aminobutyric acid isolithocholic acid (**19**), 2-aminobutyric lithocholic acid isomers (**20**, **22** and **23**), 4-aminobutyric acid lithocholic acid (**21**) (Table 2). The results successfully classified all identified MCBAs according to their conjugated amino acid or non-protein amino acid by MS/MS fragments, but only MCBAs **12**, **17**, **21** were fully confirmed by comparison with synthetized standards.

**Table 2:** MCBAs Identified and Confirmed by MS/MS Fragmentation Patterns

ID	compound name	formula	[M + H]^+^	ppm	Rt	MS/MS fragments (positive polarity)	collision E
**11**	leucolithocholic acid isomer 1^a^ ^,^ ^b^	C_30_H_51_NO_4_	490.3911	3.88	4.35	341.2839;323.2737;132.1012;**86.0957**	30
**12**	l-leucolithocholic acid^a^^,^^b^^,^^c^	C_30_H_51_NO_4_	490.3906	2.73	5.01	341.2841;323.2735;132.1018;**86.0959**	30
**13**	leucolithocholic acid isomer 2^a^	C_30_H_51_NO_4_	490.3908	1.4	5.16	132.1010;**86.0956**	30
**14**	5-aminovaloisolithocholic acid^a^	C_29_H_49_NO_4_	476.3735	0.42	3.14	458.3624;341.2821;323.2722;118.0848;**100.0743**	30
**15**	5-aminovalolithocholic acid^a^	C_29_H_49_NO_4_	476.3751	2.76	3.75	458.3650;341.2831;323.2723;118.0819;**100.0714**	30
**16**	valolithocholic acid isomer 1^b^	C_29_H_49_NO_4_	476.3746	2.45	3.95	341.2798;323.2690;118.0831;**72.0776**	30
**17**	l-valolithocholic acid^a^^,^^b^^,^^c^	C_29_H_49_NO_4_	476.3735	0.03	4.6	458.3624;341.2836;323.2734;118.0862;**72.0704**	30
**18**	valolithocholic acid isomer 2	C_29_H_49_NO_4_	476.3735	–2.05	4.77	118.0863;**72.0720**	30
**19**	4-aminobutyric isolithocholic acid	C_28_H_47_NO_4_	462.3578	–1.7	3.0	104.0689;**86.0593**	30
**20**	2-aminobutyric lithocholic acid isomer 1	C_28_H_47_NO_4_	462.3592	3.07	3.45	104.0652;**58.0599**	30
**21**	4-aminobutyric lithocholic acid^c^	C_28_H_47_NO_4_	462.3590	2.63	3.6	341.2837;323.2734;104.0700;**86.0594**	30
**22**	2-aminobutyric lithocholic acid isomer 2	C_28_H_47_NO_4_	462.3567	–2.35	4.2	341.2844;323.2734;104.0700;**58.0638**	30
**23**	2-aminobutyric lithocholic acid isomer 3	C_28_H_47_NO_4_	462.3583	1.12	4.3	104.0700; **58.0638**	30

a Detected in LCA incubation.

b Detected at t0.

c Confirmed by synthesized standard; **bold**: characteristic fragment.

Several MCBA isomer derivatives from valine and leucine, corresponding to the l and d enantiomers, were found. Although the amino group confers polarity, and previous studies reported that the enantiomer d is more polar than the l enantiomer, it was not possible to discriminate between all the valine and leucine derivatives because polarity also depends on their conjugation with isolithocholic or lithocholic acid isomers that also show a different polarity.^34^

In the case of aminobutyric acid reconjugates, many options are feasible because of the position of the amino group. This position determines the polarity of the molecule being in the polarity order α (2) > β (3) > γ (4).^35^ As for 5-aminovaleric acid, 4-aminobutyric acid has its amino group separated from the carboxylic group and not at the α carbon as in valine or 2-aminobutyric acid, and it should exhibit the most apolar behavior among the 2-, 3-, and 4-aminobutyric acids. However, upon conjugation with lithocholic acid, it retains the separation of its functional groups and facilitates possible interactions with the solvent, resulting in a higher polarity compared with that of the conjugates of 2-aminobutyric acid. This explains the lower retention time shown by the 4-aminobutyric acid conjugate compared to the valine conjugate. All MCBAs showed retention times lower than those of the free LCA backbone and therefore a higher polarity. The 4-aminobutyric isolithocholic acid conjugate (**19**) was the MCBA increasing the polarity of LCA/isoLCA the most, reducing its retention time from 5.18 to 3.0 min in the chromatograms.

The production of the MCBAs started at 48 h according to the stabilization of the production of LCA and isoLCA used as a substrate (Figure S6). All of them were continuously increasing upon 120 h except l-valolithocholic acid and 4-aminobutyric isolithocholic acid (**19**), which decreased after 72 h.

### Evaluation of Primary BAs, Secondary BAs, and MCBAs in Human Stool Samples

The BAs were analyzed in fecal samples. The primary, secondary, and conjugated BAs were identified (Table 3). The main primary BAs and secondary BAs identified were chenodeoxycholic (CDCA) (Figure 6c) and deoxycholic acids (DCA) (Figure 6d). The lithocholic acid (LCA) presented concentrations around 25-fold higher than primary BAs (Figure 6d). The deoxycholic acid was the BA more concentrated, with a mean of 9089 nmol/g in dry weight (Figure 6d). Four MCBAs were identified in the fecal sample. All of them were MCBAs based on the lithocholic core and conjugated with amino acids, two derived from valine and two derived from leucine. No MCBAs conjugated with non-proteinogenic amino acids were found in feces. The leucolithocholic acid isomer (**11**), l-leucolithocholic acid, valolithocholic acid isomer (**16**), and l-valolithocholic acid were identified as the MCBAs detected. The results showed the expected interindividual variability observed in the secondary BAs. The l-leucolithocholic acid was the only one identified across all subjects, and the leucolithocholic acid isomer (**11**) was found in nine volunteers. In the case of valine conjugates, valolithocholic acid (**16**) and l-valolithocholic acid (**17**) were found in seven volunteers.

**Figure 6:**
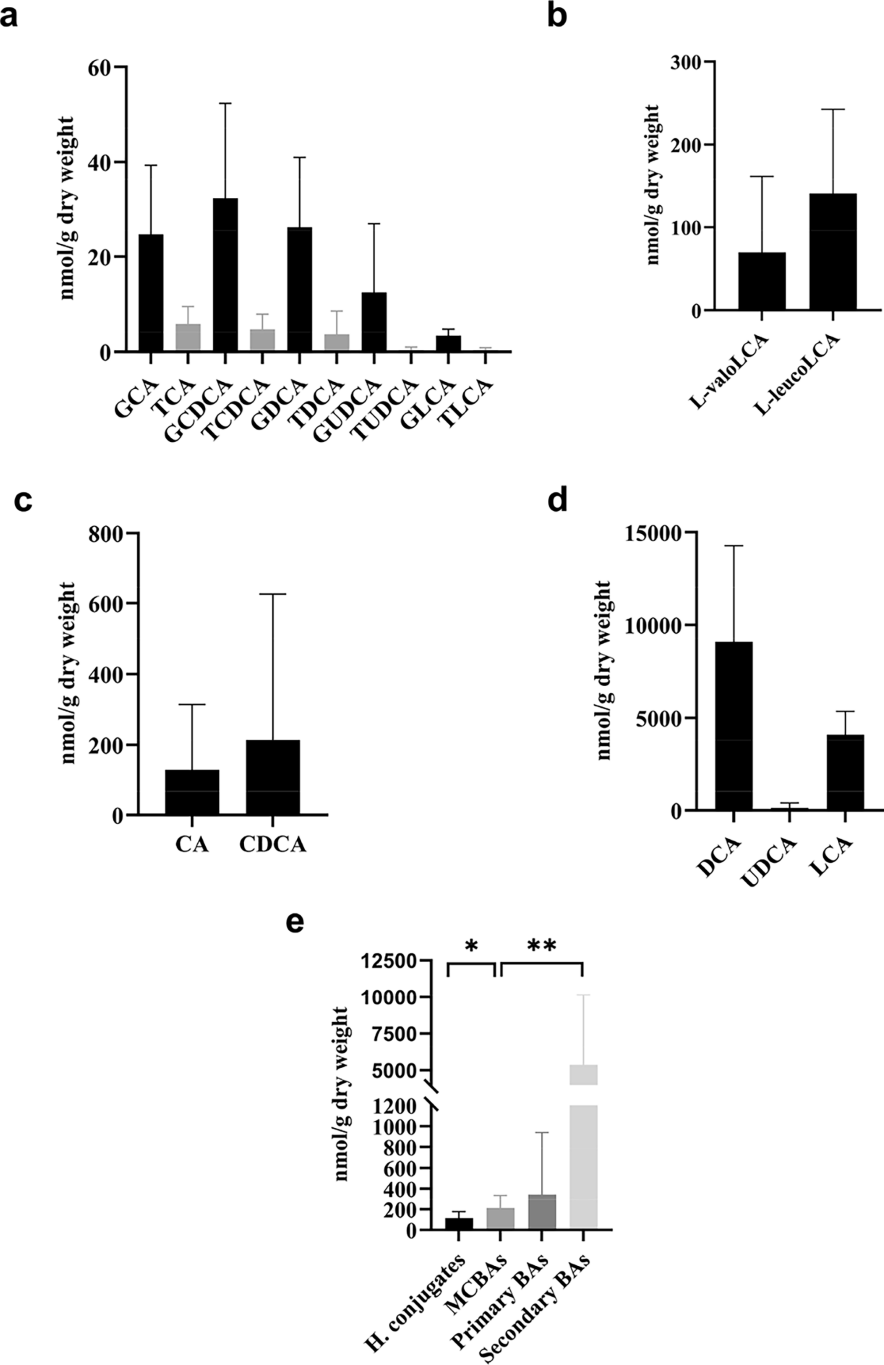
Quantification of primary BAs, secondary BAs, and MCBAs in fecal samples. *N* = 11 volunteers were included for primary and secondary and hepatically conjugated BAs (a, c, d) and *n* = 17 volunteer MCBAs (b). (a) Hepatically conjugated BAs. Glycine conjugates (black bars) and taurine conjugates (gray bars). (b) Microbially conjugated bile acids (MCBAs). l-leucoLCA, l-leucolithocholic acid (12); l-valoLCA, l-valolithocholic acid (17). (c) Primary BAs; (d) secondary BAs; (e) comparison of the total occurrence of hepatically conjugates, MCBAs, primary BAs, and secondary BAs in fecal samples. *Statistically significant (*p* < 0.05).

**Table 3:** Identification of Primary and Secondary BAs in Fecal Samples by UHPLC-QqQ-MS

ID	compound name	formula	precursor ion (*m*/*z*)	product ion (*m*/*z*)	Rt	collision E
**1**	chenodeoxycholic acid (CDCA)^a^	C_24_H_40_O_4_	451.3	391.3;373.2	4.19	28;44
**2**	lithocholic acid (LCA)	C_24_H_40_O_3_	435.31	375.2;59	8.88	16;52
**5**	ursodexycholic acid (UDCA)	C_24_H_40_O_4_	451.3	391.3;59	1.90	12;52
**25**	cholic acid (CA)	C_24_H_40_O_5_	407.28	345.3;343	2.18	32;36
**26**	cholic acid-d (CA)	C_24_H_40_O_5_	407.28	345.3;343	2.18	32;36
**27**	hyodeoxycholic acid (HDCA)	C_24_H_40_O_4_	451.3	391.3;59	2.05	12;56
**28**	glycoursodeoxycholic acid (GUDCA)	C_26_H_43_NO_5_	448.3	448.3;386.3;74	2.25	40;36;40
**29**	glycocholic acid (GCA)	C_26_H_43_NO_6_	464.3	402.3;74	2.60	36;40
**30**	taurourosdeoxycholic acid (TUDCA)	C_26_H_45_NO_6_S	498.3	498.3;124.1;80	2.73	40;60;40
**31**	taurocholic acid (TCA)	C_26_H_45_NO_7_S	514.28	124 79.9	3.02	56;60
**32**	taurocholic acid-d5 (TCA)	C_26_H_45_NO_7_S	514.28	124;79.9	3.02	56;60
**33**	deoxycholic acid (DCA)	C_24_H_40_O_4_	391.28	345.1;343.3	4.86	32;44
**34**	glycochenodeoxycholic acid (GCDCA)	C_26_H_43_NO_5_	448.3	386.3;74	5.04	36;40
**35**	glycodeoxycholic acid (GDCA)	C_26_H_43_NO_5_	448.3	402.3;74	5.74	40;36
**36**	taurochenodeoxycholic acid (TCDCA)	C_26_H_45_NO_6_S	498.3	498.3;498.3	5.97	60 60
**37**	taurodeoxycholic acid (TDCA)	C_26_H_45_NO_6_S	498.3	124.1;79.9	6.63	60;60
**38**	glycolithocholic acid (GLCA)	C_26_H_43_NO_4_	432.3	74	9.48	40
**39**	taurolithocholic acid (TLCA)	C_26_H_45_NO_5_S	482.29	124;80	2.73	56;60

a a

The presence of the hepatic conjugates based on glycine was significantly much higher than taurine. The occurrence of the hepatic conjugated primary and secondary BAs (Figure 6a) was around three times lower than that of MCBAs (Figure 6b). Only the l-leucolithocholic acid and l-valolithocholic acid were quantified using standards, and the mean concentrations were 140 and 70 nmol/g of dry weight, respectively. The occurrence of MCBAs in fecal samples was significantly higher than that of hepatically conjugated bile acids, being approximately 3-fold greater (Figure 6e). This is the first time that the relevance of these conjugates has been reported in comparison to hepatic conjugates. Moreover, it should be noted that only two MCBAs were quantified in this study, which may represent only a fraction of their total relevance. The results demonstrated the importance of microbial conjugates and the need to take them into consideration in BAs analysis.

## Discussion

The interaction between the gut microbiota and the BAs is a crucial point in evaluating the relationship of BAs with intestinal receptors and their implications in metabolic disorders. This study of fecal incubation with the primary BA, CDCA, showed the global trend of microbial BA metabolism to produce mostly epimers of chenodeoxycholic acid, lithocholic acid, and isolithocholic acid, accumulating these last two. The mechanism to produce β-orientation of the hydroxyl groups confers more hydrophobicity to the molecule and therefore reduces its toxicity.^36^ The hydroxysteroid dehydrogenase (HSD) is the enzymatic activity capable of performing it and is carried out by the bacterial enzymes that act on the hydroxyl groups of the BAs. Additionally, the BA hydrophobicity and therefore toxicity depend on the number of the hydroxyl groups, with LCA being the most hydrophobic BA.^37^ The results showed LCA as the major BA backbone substrate to produce the MCBAs. These results suggested a possible specific requirement of the bacteria to avoid the presence of the unconjugated LCA that has been described as highly toxic.^38^ This study suggested this mechanism for the first time. This is similar to what happens with hepatically conjugated BAs. In general, the occurrence of primary BAs conjugated with glycine and taurine confers on them specific physicochemical and metabolic properties for preventing their toxicity. These conjugates increase the solubility in acidic pH and are fully ionized at small intestine pH. This prevents the passive absorption of the epithelial cell and passive paracellular absorption by the size and negative charge of the molecule. If BAs were synthetically conjugated with other amino acids, then such conjugates would be readily hydrolyzed by pancreatic carboxypeptidases.^39^ Therefore, conjugation with glycine and especially taurine makes BAs hardly absorbable and ensures their role as detergent in lipids absorption. Unconjugated and some glycine-conjugated BAs are reabsorbed via passive diffusion along the small intestine; on the contrary, the active transport of BAs occurs in the ileum while passive absorption of hydrophobic secondary BAs occurs in the colon.^40^

Additionally, the gut bacteria are able to hydrolyze the conjugated BAs and also produce the MCBAs by the BSH activity, and they are widespread in commensal bacteria colonizing both the small intestine and colon.^41^ The BSH activity and, therefore, the balance of unconjugated/conjugated BAs may play a key role by improving the control of different metabolic disorders related to cholesterol, lipid, and glucose metabolism, bowel disease, or cancer.^22–30^ It has been described how the modulation of the BSH activity increases the ileum content of conjugated BAs inhibiting the intestinal FXR-FGF15 signaling pathway and leading to a reduction of the hepatic cholesterol and decreases lipogenesis. The aforementioned relevance of the unconjugated/conjugated BA ratio and their ability to be absorbed show the importance of the new MCBAs and the need for adequate characterization. This suggests conducting future studies focused on evaluating how this balance affects states of intestinal inflammation or the interaction with intestinal receptors in specific animals and cell cultures.^42^

This study confirmed 13 MCBAs based on AAs and non-proteinogenic AAs and characterized for the first time the fragmentation rules to identify and differentiate the structural isomers. In this way, it has been possible to differentiate, for example, between MCBAs formed with valine and 5-aminovaleric acid, and 4-aminobutyric and 2-aminobutyric acids, which exhibit the same *m*/*z* but have the amino group in different positions. These compounds had previously been erroneously classified as valine derivatives or esterified MCBAs. MCBAs conjugated with nitrogenous acids containing the amino group on the α carbon, as proteinogenic AAs and 2-aminobutyric, release a fragment corresponding to water plus carbon monoxide loss (−46), while nitrogenous acids containing a terminal amino group, as in 5-aminovaleric acid or 4-aminobutyric acid, release a fragment corresponding to the water loss (−18). The results of the study showed a polarity reduction of the MCBAs compared with that of the lithocholic acid, especially the aminobutyric lithocholic acid (9). These results suggest that this reconjugation could make difficult the passive absorption of unconjugated and more hydrophobic secondary BAs.

Furthermore, the results indicated that lithocholic acid–based MCBAs were the most relevant both after the *in vitro* incubation and in the fresh stool samples. These findings are relevant since lithocholic acid has been identified as a calorie restriction-induced metabolite that alone recapitulates the antiaging benefits of CR by activating AMPK, enhancing muscle regeneration, improving physical performance, and extending health span and lifespan in an AMPK-dependent manner across multiple model organisms.^43^ The identified MCBAs were conjugates with branched-chain amino acids (BCAA) and non-proteinogenic amino acids as 5-aminovaleric acid and aminobutyric acid derivatives. The link between the gut microbiota and the abundance of the BCAA levels and insulin resistance has been studied showing an increase of BCAA in studies where the obesity and diabetes model were evaluated.^44^ We hypothesized that nonhealthy individuals, because of the gut dysbiosis, are not able to produce the reconjugated derivatives with the BCAA and show an increase of free BCAA in the fecal metabolome. Regarding aminobutyric acid derivatives, some strains of bacteria have demonstrated the ability to produce gamma-aminobutyric acid (GABA) from glutamate in the human intestinal tract.^45,46^ Therefore, the reconjugation of BAs with aminobutyric acid derivatives may be feasible. The possibility of being able to differentiate structural isomers using fragmentation rules represents a great advance given the relevance of GABA compared to 2-aminobutyric acid, or even misidentify valine conjugates with those of 5-aminovaleric acid, which is an agonist of GABA. In addition, the role of 5-aminovaleric acid as a precursor of 5-aminovaleric acid betaine, which is a compound associated with the gut microbiota with positive health effects such as fetal brain development, insulin secretion, and reduced cancer risk, has been studied. However, it has also been linked with some negative health outcomes such as cardiovascular disease and fatty liver disease.^47^ There is no evidence yet that its conjugation with BAs is associated with positive or negative effects, but there is definitely a need to study these new molecules.

Lastly, this study analyzed and quantified the primary and secondary BAs (both conjugated and unconjugated) as well as the newly identified MCBAs in fecal samples. The results revealed that the concentration of MCBAs was approximately 3-fold higher than that of the hepatically conjugated BAs. Here, only two of the hundreds of possible reconjugation products were identified and quantified in the *in vivo* study, compared to 13 detected in the *in vitro* study. However, this may serve as a starting point for future investigations, particularly in studies involving groups with metabolic disorders related to cholesterol, lipids, or glucose. This finding underscores the relevance of these newly identified microbial conjugates, highlighting the necessity of incorporating them into standard BA analytical protocols.

Finally, the biological relevance of these newly identified MCBAs remains to be determined, as their potential may lie in balancing the levels of conjugated bile acids and modulating their interactions with host receptors, in preventing free lithocholic acid from exerting its biological effects or being transported more efficiently, and even in acting as scavengers or mobilizers of other highly bioactive molecules, such as GABA.^48,49^ Overall, the significance of the BAs in lipid and glucose metabolism supports the need to develop suitable methods to evaluate them. In particular, the MCBAs produced by the gut microbiota, little studied so far, is an important step for full knowledge of the BA profile. In addition, the differentiation between structural isomers of MCBAs could open new opportunities to evaluate individually the impact of the BAs metabolism on health.

## Supplementary Material



## Data Availability

Raw metabolomics data of the present study are available at ZENODO repository (URL: 10.5281/zenodo.14450057)

## Notes

*In vivo* and *in vitro* study, MCBA extraction method, MCBA fragmentation, and primary and secondary BAs (PDF)

## Notes

A first version of this manuscript was deposited as a preprint at BioRxiv. DOI: 10.1101/2023.11.07.564921.
