# Supplementary material for: Novel Insights into the Human Gut Microbially Conjugated Bile Acids: The New Diversity of the Amino Acid-Conjugated Derivatives
Source: J Agric Food Chem. 2025 Jul 24;73(31):19460–73. doi: 10.1021/acs.jafc.5c03548 (PMC12333354; doi:10.1021/acs.jafc.5c03548)

Supporting information

Novel insights into the human gut microbially conjugated bile acids: the new diversity of the amino conjugated

Carlos J. Garcia[a*], Rocio García-Villalba[a], Maria D. Frutos-Lisón[a], David Beltrán[a], María Antonia Martínez-Sánchez[b], María Ángeles Núñez-Sánchez[b], Bruno Ramos-Molina[b], Francisco A. Tomás-Barberán[a]

[a]Quality, Safety and Bioactivity of Plant-Derived Foods, Centro de Edafología y Biología Aplicada del Segura-Consejo Superior de Investigaciones Científicas (CEBAS-CSIC), 30100 Murcia, Spain;

[b] Obesity, Diabetes and Metabolism Research Group, Biomedical Research Institute of Murcia-Pascual Parrilla (IMIB-PP, 30120 Murcia, Spain

*Corresponding author: Phone: +34-968396200 (ext. 445454); E-mail address: cjgarcia@cebas.csic.es (C. J. G)

Supplementary Figures	Content
1	Scheme of the analysis of bile acids by the different instruments
2	BAs analysis workflow
3	MS/MS spectra of MCBAs isomers with valine and 5-aminovaleric acid in positive polarity
4	MS/MS spectra of MCBAs isomers with leucine in positive polarity
5	MS/MS spectra of MCBAs isomers with aminobutyric acid derivatives in positive polarity
6	Production kinetics of MCBAs during 120 h

Supplementary Figure 1. Scheme of the analysis of bile acids by the different instruments

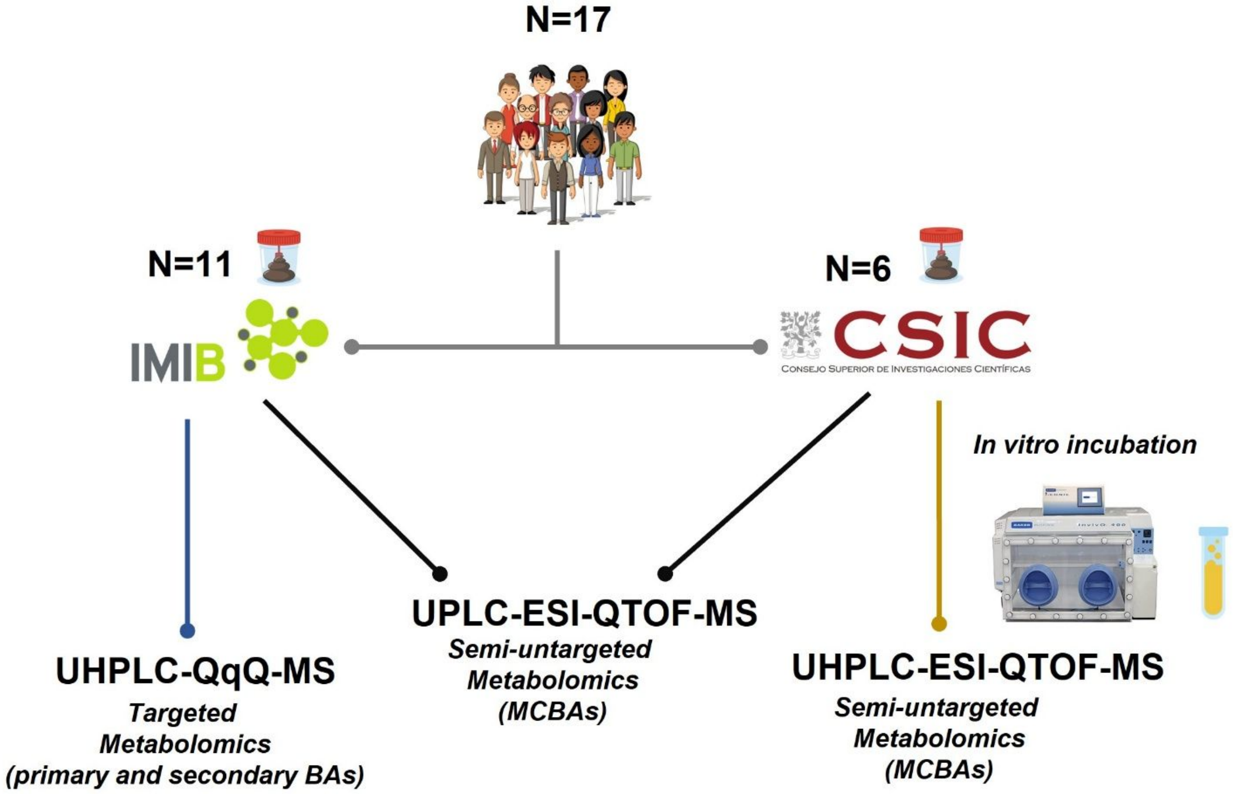

Supplementary Figure 2. BAs analysis workflow. 1) Fresh fecal samples collected by each volunteer; 2) Dilution 1/10 of the sample in the medium and mixture by stomacher for 3 minutes; 3) Incubation tubes including: medium + total fecal bacteria (a), medium + total fecal bacteria + BAs (b); 4) Tubes were incubated for 5 days at anaerobic conditions; 5) After each sampling point samples were centrifuged and supernatant collected; 6) SPE extractions were performed for BAs concertation; 7) The eluates were speed vacuum dried and re-dissolved in 100 µL of MeOH prior UPLC-QTOF-MS analyses

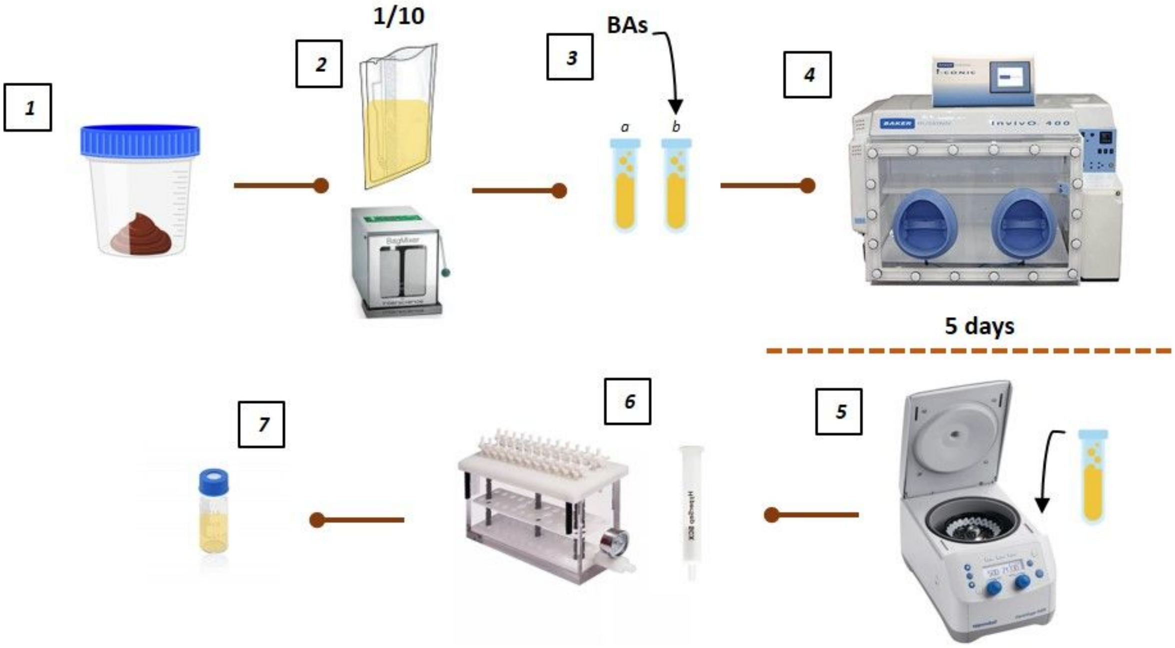

Supplementary Figure 3. MS/MS spectra of MCBAs isomers with valine and 5-aminovaleric acid in positive polarity. Nitrogenous acids with a terminal amino group (red MS/MS spectra), nitrogenous acids with the amino group at the alpha carbon (blue MS/MS spectra). Characteristic fragments of the nitrogenous acid (grey circle); characteristic fragment of the H(2)O loss related to nitrogenous acids with terminal amino group (orange circle); characteristic fragment of H(2)O plus CO loss related to nitrogenous acids with amino group at the alpha carbon (green circle). 5-Aminovaloisolithocholic acid **(14)**; 5-Aminovalolithocholic acid **(15)**; Valolithocholic acid isomer 1 **(16)**; L-Valolithocholic acid **(17)**; Valolithocholic acid isomer 2 **(8)**.

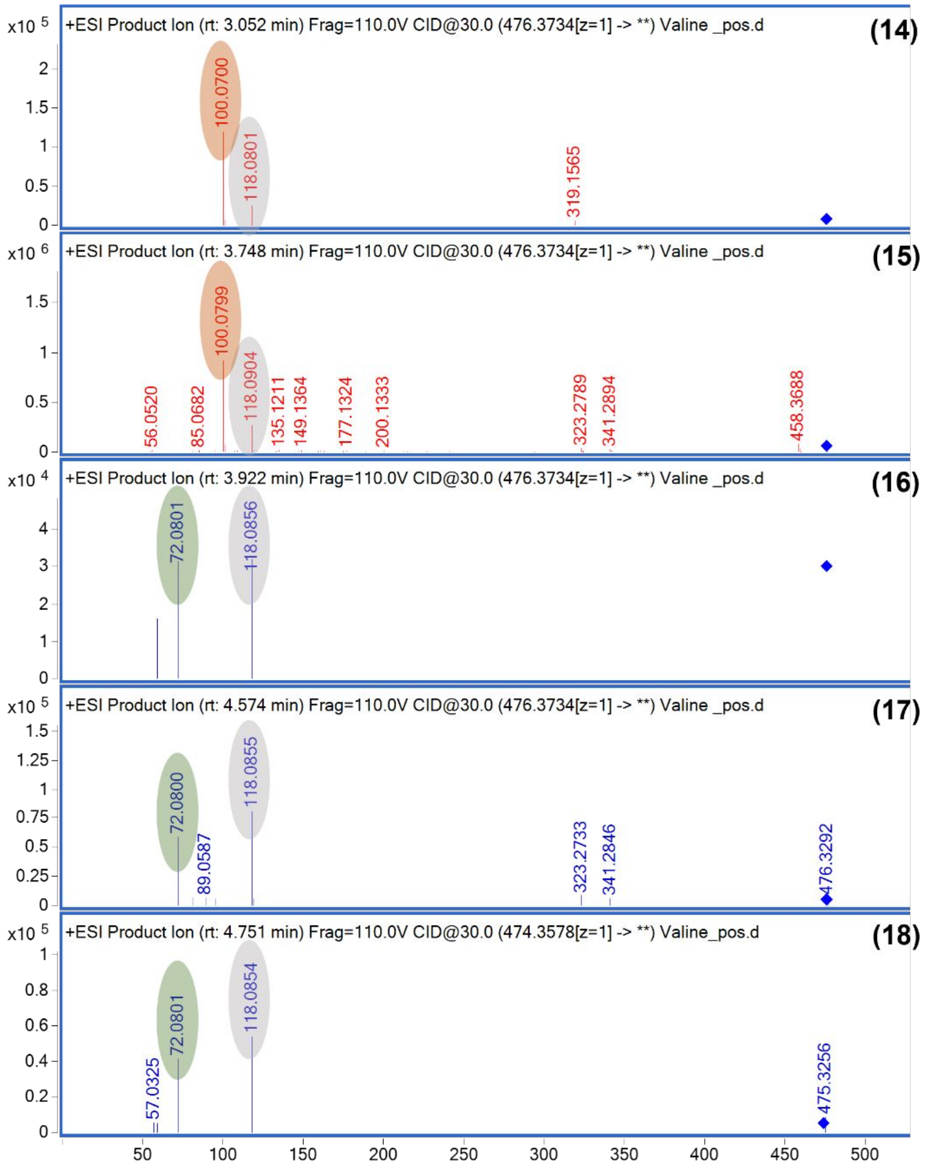

Supplementary Figure 4. MS/MS spectra of MCBAs isomers with leucine in positive polarity. Characteristic fragment of the leucine with amino group at alpha carbon (grey circle); characteristic fragment of H(2)O plus CO loss (green circle); Leucolithocholic acid isomer 1 **(11)**; L-Leucolithocholic acid **(12)** ; Leucolithocholic acid isomer 2 **(13)**.

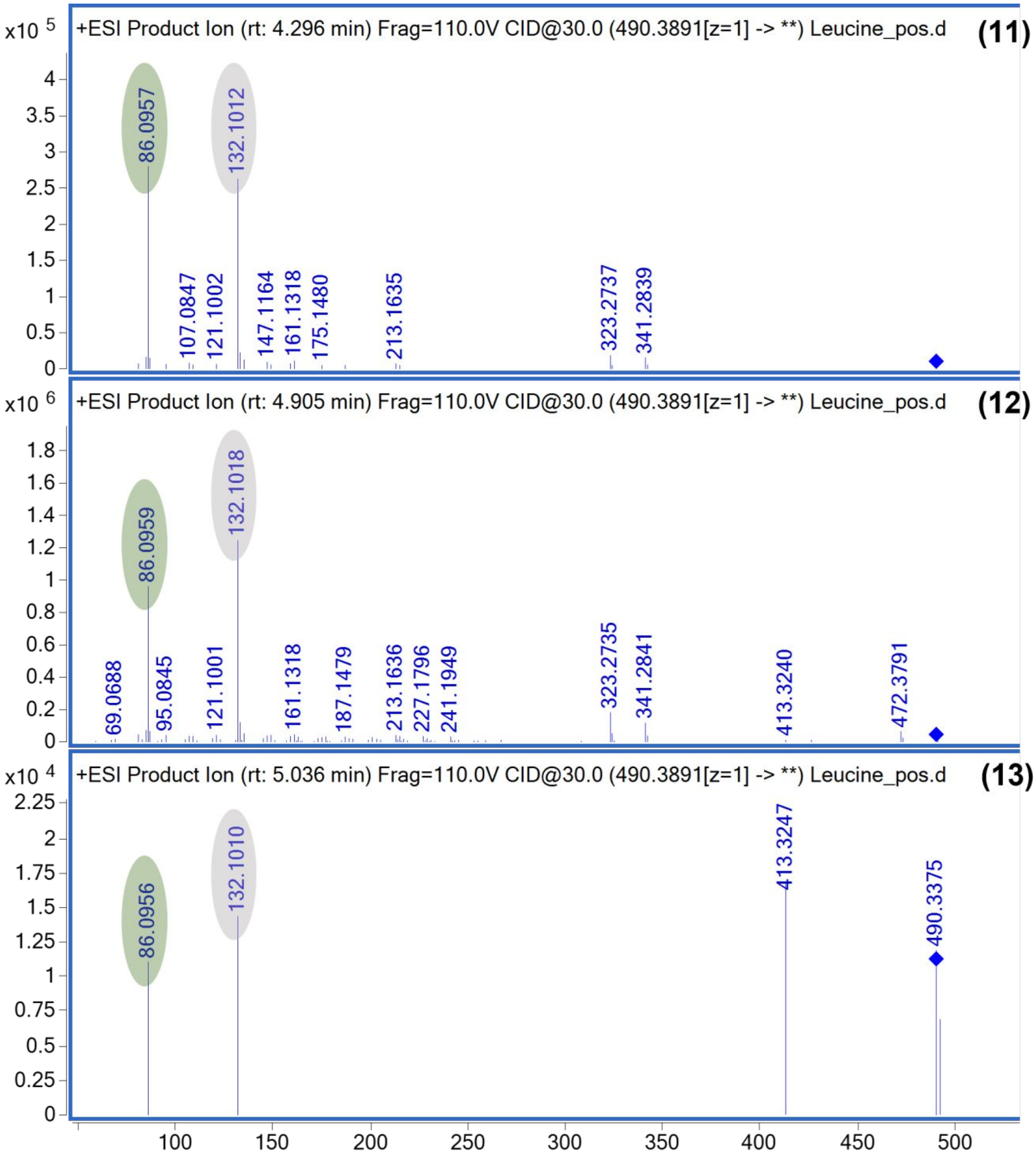

Supplementary Figure 5. MS/MS spectra of MCBAs isomers with aminobutyric acid derivatives in positive polarity. Nitrogenous acids with terminal amino group (red MS/MS spectra), nitrogenous acids with the amino group at alpha carbon (blue MS/MS spectra). Characteristic fragment of the aminobutyric acid derivatives (grey circle); characteristic fragment of the H(2)O loss related to nitrogenous acids with terminal amino group (orange circle), characteristic fragment of H(2)O plus CO loss related to nitrogenous acids with amino group at the alpha carbon (green circle). 4-Aminobutyric isolithocholic acid **(9)**; 2-Aminobutyric lithocholic acid isomer 1**(10)**; 4-Aminobutyric lithocholic acid **(11)**; 2-Aminobutyric lithocholic acid isomer 2 **(12)**; 2-Aminobutyric lithocholic acid isomer 3 **(13)**.

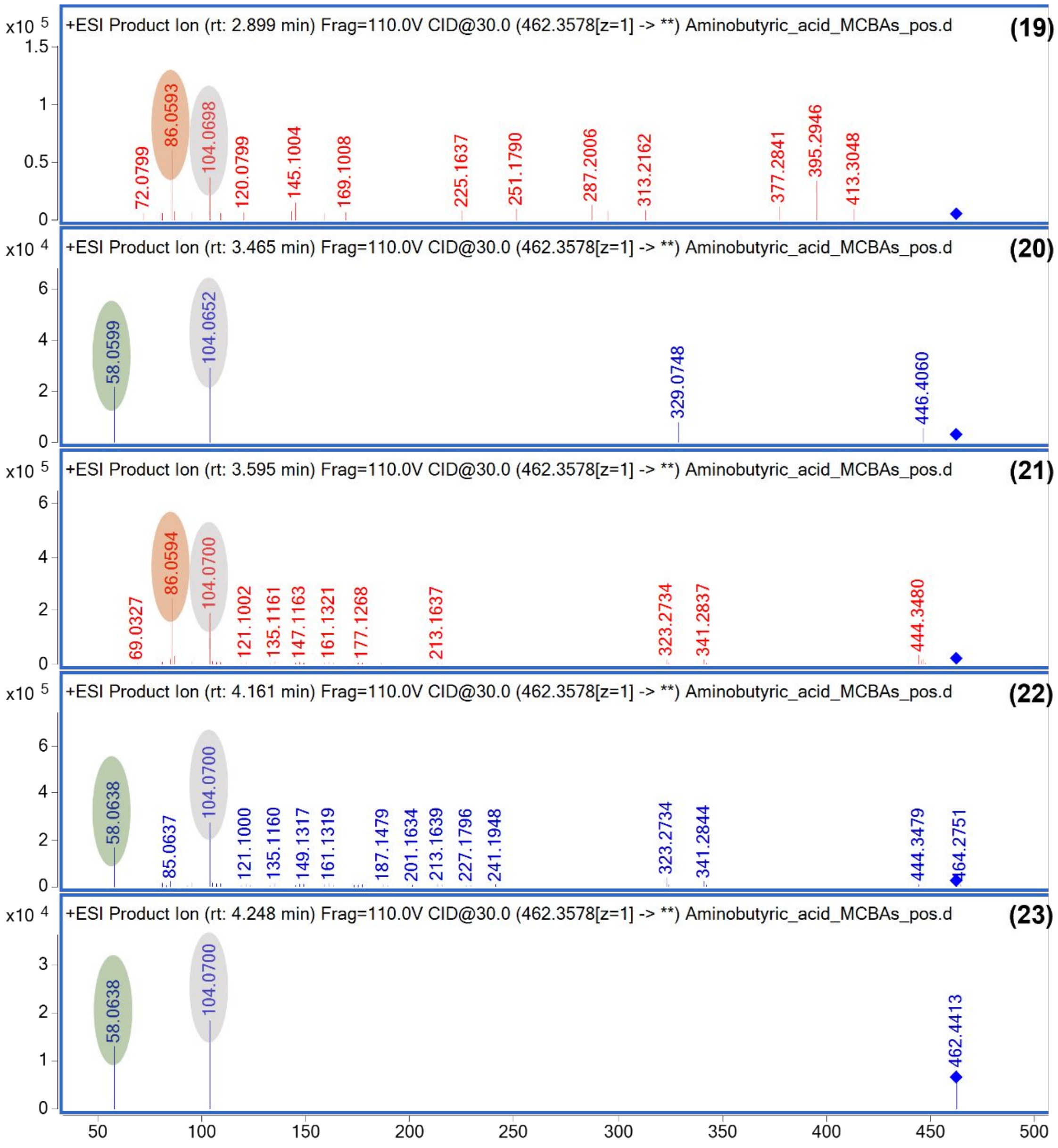

Supplementary Figure 6. Production kinetics of MCBAs during 120 h. MCBAs graphs: 1) Leucolithocholic acid isomer **(11)**; 2) L-Leucolithocholic acid **(12)**; 3) Leucolithocholic acid isomer **(13)** ; 4) 5-Aminovaloisolithocholic acid (**14**); 5) 5-Aminovalolithocholic acid **(15)**; 6) Valolithocholic acid isomer **(16)**; 7) L-Valolithocholic acid **(17)**; 8) Valolithocholic acid isomer **(18)**; 9) 4-Aminobutyric isolithocholic acid **(19)**; 10) 2-Aminobutyric lithocholic acid isomer **(20)** ; 11) 4-Aminobutyric lithocholic acid **(21)**; 12) **(22)** 2-Aminobutyric lithocholic acid isomer **(22)**; 13) 2-Aminobutyric lithocholic acid isomer **(23)**

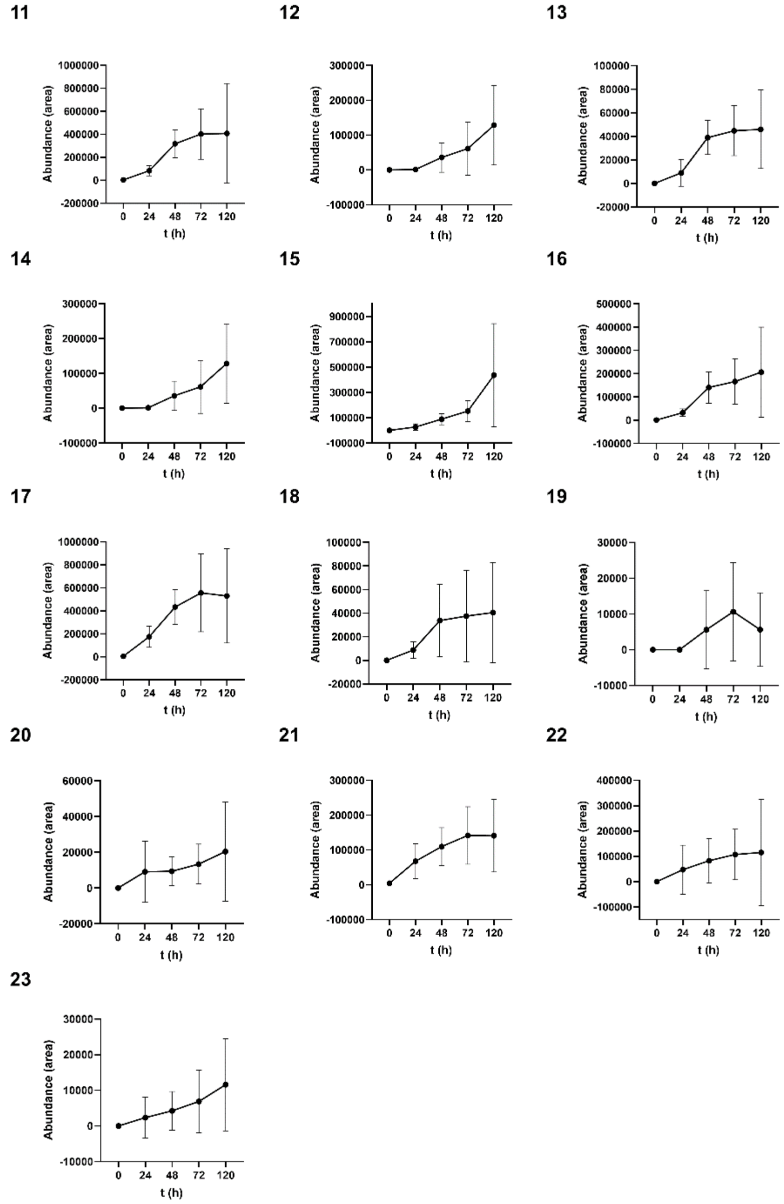

Supplement: Supplementary file 1 [file jf5c03548_si_001.pdf]
